# Insights into the production and evolution of lantibiotics from a computational analysis of peptides associated with the lanthipeptide cyclase domain

**DOI:** 10.1098/rsos.240491

**Published:** 2024-07-17

**Authors:** Nikunj Maheshwari, Lars S. Jermiin, Chiara Cotroneo, Stephen V. Gordon, Denis C. Shields

**Affiliations:** ^1^ School of Medicine, University College Dublin, Dublin, Ireland; ^2^ Conway Institute of Biomolecular and Biomedical Research, University College Dublin, Dublin, Ireland; ^3^ Research School of Biology, Australian National University, Canberra, ACT, Australia; ^4^ School of Mathematical and Statistical Sciences, University of Galway, Galway, Ireland; ^5^ School of Veterinary Medicine, University College Dublin, Dublin, Ireland

**Keywords:** antibiotic, lanthipeptide, lantibiotic, evolution, cyclic peptide, bridging, structure, cyclase

## Abstract

Lanthipeptides are a large group of ribosomally encoded peptides cyclized by thioether and methylene bridges, which include the lantibiotics, lanthipeptides with antimicrobial activity. There are over 100 experimentally characterized lanthipeptides, with at least 25 distinct cyclization bridging patterns. We set out to understand the evolutionary dynamics and diversity of lanthipeptides. We identified 977 peptides in 2785 bacterial genomes from short open-reading frames encoding lanthipeptide modifiable amino acids (C, S and T) that lay chromosomally adjacent to genes encoding proteins containing the cyclase domain. These appeared to be synthesized by both known and novel enzymatic combinations. Our predictor of bridging topology suggested 36 novel-predicted topologies, including a single-cysteine topology seen in 179 lanthionine or labionin containing peptides, which were enriched for histidine. Evidence that supported the relevance of the single-cysteine containing lanthipeptide precursors included the presence of the labionin motif among single cysteine peptides that clustered with labionin-associated synthetase domains, and the leader features of experimentally defined lanthipeptides that were shared with single cysteine predictions. Evolutionary rate variation among peptide subfamilies suggests that selection pressures for functional change differ among subfamilies. Lanthipeptides that have recently evolved specific novel features may represent a richer source of potential novel antimicrobials, since their target species may have had less time to evolve resistance.

## 1. Introduction

Bacteriocins [1] are bacterial ribosomally produced peptide antimicrobials that modulate the dynamics of microbial populations [2] by controlling the growth of other species [3,4]. Computational surveys of genomes can pinpoint genes encoding bacteriocins, such as may be performed with the BAGLE3 software [5]. Sets of bacteriocin predictions can be considered as a virtual library of potential compounds that could be mined for antibiotics or other functional activities. It is of interest to characterize the peptide sequence space occupied by such a virtual library [6], to determine the range and diversity of predicted sequences, and to place an understanding of diversity in the context of both evolutionary and functional constraints. Such an approach can help identify regions of the peptide space that are underexplored and would benefit from more experimental investigation.

To gain insights into the nature of predicted bacteriocin sequence diversity, a good starting point is an extensive bacteriocin family, the ribosomally synthesized lantibiotics [7]. These are antimicrobial lanthipeptides whose cyclic modified amino acids lanthionine (Lan) and/or β-methyl lanthionine (MeLan) [8] comprise a thioether bridge between a cysteine and a dehydrated serine or threonine [9,10]. Alternatively, some form a labionin modification, which is a cyclic structure derived from cysteine and two preceding dehydrated serines [11]. Typically, the precursor’s leader peptide is recognized by the lantibiotic dehydratase–cyclase–transporter complex [12]. The dehydratase [13] and cyclase [14] generate Lan/MeLan bridges, and then the modified precursor may be transported across the lipid bilayer [15,16] where peptidase cleavage [17,18] yields the active antimicrobial peptide, or the precursor may alternatively be removed prior to transport across the membrane [16].

Thus, lanthipeptide cyclization is governed by two key enzymatic steps encoded by dehydratase and cyclase [19] domains. Genes encoding these domains typically cluster chromosomally with the precursor peptide’s gene. The vast majority of cyclizations are performed by the LanC-like protein domain, named after the LanC protein in *Lactobacillus lactis* [14]. Different combinations of LanC-like cyclase subtype and dehydratase alternative enzymes are classified into five synthetase types, I, II, IIa, III and IV [20]. Type III cyclases act on SxxSx{2,5}C motifs (where S is serine, C is cysteine, x indicates any amino acid and x{2,5} indicates between two and five residues). These generate either a bicyclic labionin structure linking those three residues via both lanthionine (cysteine–serine) and methylene (serine–serine) bridges, or a lanthionine thioether bridge linking the cysteine to only one serine [21]. Type I, II, IIa and IV synthetases act on [ST]*C motifs (here, * represents at least two residues), or occasionally C*[ST] motifs, to introduce lanthionine or methyllanthionine bridges of cysteine to serine or threonine, respectively [22]. Thus, evolution has favoured multiple alternative dehydratases, with three unrelated domains (LanB, DUF4135, Lyase; table 1) identified to date that can perform this role [23].

**Table 1.** Nomenclatures and number of predicted lanthipeptides clustering closest to each synthetase configuration^a^.

		dehydratase domain^b^		
		B, LanB	D, DUF4135	L, Lyase
**cyclase^c^**	**CCH**	Type I, B-CCH, 194	Type II, D-CCH, 221	Type IV, L-CCH, 33
	**CCC**	B-CCC, 2	Type IIa, D-CCC, 79	L-CCC, 8
	**unknown**	B-Unk, 14	D-Unk, 47	Type III, L-Unk, 192

^a^ Cyclase triad and dehydratase with colours as used in figure 1; where types are previously classified [22], the type is shown in addition to the naming convention of this analysis.

^b^ Dehydratase domains: B, LanB: LanB-related dehydratase; D, DUF4135: PFAM DUF44135-related dehydratase.

^c^ Cyclase: CCH corresponds to cyclase with zinc-binding triad residues cysteine, cysteine, histidine; CCC to cysteine, cysteine, cysteine and unknown (Unk) corresponds to a cyclase with an unknown catalytic mechanism.

**Figure 1.**
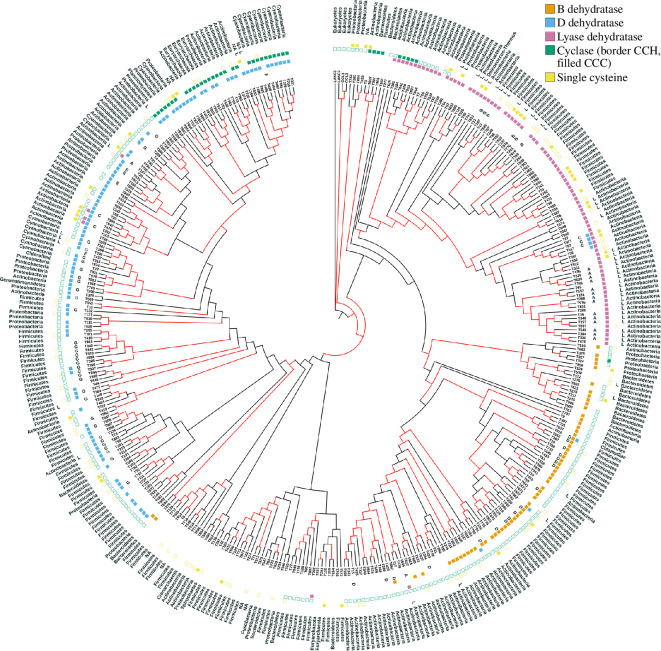
Cyclase domain phylogenetic tree. From centre: phylogenetic tree, with an eukaryotic root at 12 o'clock, red indicates branches with >90% bootstrap support; codes starting T identify a predicted peptide clustering with the cyclase (see electronic supplementary material, dataset 2); letters A–G: peptide subfamilies; orange, blue and pink: B, D and L dehydratases; cyclase filled green: CCC triad, border green: CCH, blank: unknown; yellow filled: only single cysteine peptide(s) predicted in the cluster, yellow border: cluster includes a single cysteine as well as other peptide(s); L: peptide with labionin motif; bacterial phylum. An alternative rectangular layout of this tree is provided in electronic supplementary material, figure S1 to complement the circular visualization.

Lanthipeptide biosynthesis genes typically cluster close to the lanthipeptide precursor’s (LP’s) open reading frame (ORF), assisting the computational mining of bacterial genomes for lanthipeptide sequences [5] and leading to the discovery and experimental validation of lantibiotics, such as pneumococcin [24] and cerecidins [25]. The positioning of bridges appears to be determined largely by the precursor sequence, and not by differences among synthetases [26]. This makes it potentially feasible to predict the bridging pattern (topology) among multiple cysteines and serine/threonines, based on the sequences of peptide precursors, although to date, no such predictors have been proposed. Here, we surveyed 2785 bacterial genomes to characterize the evolutionary diversity of both synthetases and predicted lanthipeptides, and their predicted peptide bridging topologies. This identified predicted topological diversity beyond that represented among experimentally characterized lanthipeptides, and it suggests an ancient origin for lanthipeptides.

## 2. Material and methods

### 2.1. Identification of candidate lanthipeptide ORFs adjacent to cyclase domains

A total of 2785 complete sequenced bacterial genomes were obtained from NCBI. Prodigal [27] was used (with default parameters) to find ORFs in the genomes downloaded from NCBI. These ORFs were searched (Expectation value < 0.01) using Hidden Markov Models (HMMs) representations of relevant domains (electronic supplementary material, table S1) by HMMer 3.1b2 [28]. PFAM (Protein FAMily) domains (PF) (May 2015; electronic supplementary material, table S1) [29] were used to search for lantibiotic modification and processing enzymes. Kinase (PF00069) and lyase are the two components of type III/IV synthetases that catalyse dehydration. The Lyase HMM was prepared as follows: (i) the first 170 residues of VenL (UniProt F2R8I9) were searched with BLASTP [30] in the non-redundant NCBI protein database and (ii) matching regions were aligned using Clustal Omega [31], and the alignment searched with HMMsearch [28] versus the reference proteomes. The aligned matches (electronic supplementary material, figure S6) were used to build an HMM using HMMbuild [28].

We used ORFs of less than or equal to 100 amino acids (the largest known lantibiotic precursor for cinnamycin is 78 residues long [32]), with one or more cysteines, where the number of serine and threonine residues was greater than or equal to the number of cysteine residues. We also required that the last cysteine residue was located within the last third of the peptide, in order to reduce the number of false-positive peptides, where the only cysteine was in the region more typically occupied by the leader peptide. Candidate precursors were selected according to the following criteria: they needed to lie within a cluster of one or more genes encoding proteins with lanthipeptide gene cluster-associated domains, and the cluster needed to include a match to the cyclase domain. A cluster was defined as a set of proteins matching one or more of the 15 PFAM domains (electronic supplementary material, table S1) or precursors, where the distance between any adjacent matching domains/precursors does not exceed 10 kb. The 15 protein domains represent a cyclase domain, seven dehydratase-associated domains, two peptidase domains, a transporter domain and four two-component system domains (electronic supplementary material, table S1).

Synthetase combinations were assigned to all precursor ORFs that lay in gene clusters which had, in addition to the cyclase domain, the domains associated with a particular dehydratase. In the case of a small number of clusters containing multiple dehydratase types, the precursor ORF was assigned to the nearest dehydratase domain.

#### 2.1.1. Homology and evolutionary analysis

Each inferred precursor was aligned to known lantibiotics using ggsearch36 global alignment [33]. To correct compositional biases leading to incorrect inference of homology, each precursor was searched against a database of known lantibiotic sequences with the leader peptide internally randomized, and the core peptide internally randomized. With an expectation of such a match by chance (*E*-value) of 10^−15^, only 1 in 1000 precursors matched a randomized sequence (false-discovery rate of 0.001), so we considered matching precursors with *E*-values ≤10^−15^ as likely homologues.

Cyclase domain sequences were aligned using MAFFT [34] in Ginsi mode. Sequences found to be random with respect to other sequences were identified using SatuRation v. 1.0 (www.github.com/lsjermiin/SatuRation.v1.0) and SatuRationHeatMapper (www.github.com/ZFMK/SatuRationHeatMapper) and removed. This two-step procedure of alignment and removal of dubious sequences was repeated until none of the sequences in the smaller alignment were considered dubious. Sites in this 389-sequence alignment with less than 50% unambiguous characters (i.e. A, C, D, E, F, G, H, I, K, L, M, N, P, Q, R, S, T, V, W and Y) were removed using AliStat v. 1.14 [35], creating a final alignment with 673 sites and a completeness score of 71.5%, up from 18.3% before the alignment was masked. The optimal model of sequence evolution for the final alignment was identified using ModelFinder [36] and the BIC (Bayesian68 Information Criterion) optimality criterion. Using this model (i.e. Q.pfam+FO+I+R9), the optimal tree, with bootstrap scores inferred using the UFBoot2 method [37], was inferred using IQ-TREE2 v. 2.2.0.5 [38]. The optimal phylogenetic estimate was visualized using iTol v. 3 [39,40].

We retrieved a 16S rRNA tree of bacterial strains [41]. Using the ETE (Environment for Tree Exploration) toolkit [42], the tree was pruned to retain species of interest (1026 nodes). For each species, individual strains were added with an interstrain distance = 0 (2139 nodes). Phylogenetic distances of cyclase domains and 16 s rRNA were calculated using Dendropy [43].

#### 2.1.2. Sequence analysis

Motifs in precursors were predicted using SLiMFinder (Short linear motif finder) 5.2 [44]. Charge and hydrophobicity were calculated using Bioperl [45].

To analyse compositional effects, LPs were redundancy-reduced by 50% identity using CD-HIT (Cluster Database at High Identity with Tolerance) [46] yielding 483 non-similar lanthipeptides. For each, two length-matched genomically encoded true negatives were selected as ORF precursors matching the sequence rules but located outside the lanthipeptide clusters. For each amino acid in a peptide (and for hydrophobicity and net charge), we calculated the fractional position (residue position/length), whose correlation was then calculated with the amino acid’s presence or absence (1/0). In clusters with multiple cyclases, the predicted precursor was assigned to the chromosomally closest cyclase .

A rule-based predictor of bridging topology was implemented in perl (see electronic supplementary material compressed file of code for cluster identification and bridge prediction). The scripts were written in Perl v. 5.18.2 and BioPerl 1.6.923, and tested on Linux Ubuntu 16.04 LTS. Given the small size of the training dataset of known lanthipeptides, we chose not to rely on complex prediction tools such as machine learning, which can easily overfit and as a consequence overestimate the confidence in their predictions. We relied on a more readily interpretable set of simple rules that accounted for the majority of known bridging patterns. The chief advantage of a rule-based predictor is that it is easy to understand how the rules were applied, and there is a clearer understanding of its limitations in particular contexts.

## 3. Results

### 3.1. Precursor lanthipeptide identification

Our goal was to develop a method that could survey beyond the set of lanthipeptides that have significant similarity with previously identified lanthipeptides, or with similar dehydratase associations. A dataset of 2785 complete bacterial genomes (electronic supplementary material, table S2) were searched for short ORFs of up to 100 amino acids adjacent to cyclase genes, with at least one cysteine and sufficient corresponding serines and threonines to allow the cysteines to form Lan/MeLan or labionin bridges. A total of 918 uncharacterized LPs were predicted in addition to 33 known lanthipeptides. Many experimentally known lantibiotics were not predicted, largely because the full genome sequence of their strain was not available in the survey dataset at the time of download. Precursor peptides were from 29 to 100 amino acids long, with certain core peptides (suggested by cleavage site and leader properties) likely to range from 7 to 53 peptides in length.

### 3.2. Predicted peptides associated with novel synthetases

A single known protein domain is implicated in most lanthipeptide cyclization and is found in both Gram-positive and Gram-negative bacteria [47,48]. There are three main functional and evolutionary groupings of the cyclase domain. Here, we name these CCH and CCC (with Cys, Cys, His and Cys, Cys, Cys, respectively, in their key zinc-binding triad residues; sometimes referred to as a CHG versus CCG motif difference, since two of the triad residues are adjacent in the sequence, and they are followed by a conserved glycine [23]) and ‘Unk’, in which the triad residues are not defined. There are three unrelated known dehydratase domains, here denoted B (LanB-related dehydratase [49]), D (DUF4135-related dehydratase [50]) and L (lyase-related dehydratase, always coupled with a kinase [48]). Known lanthionine synthetases are classified [22] as Types I (B-CCH), II (D-CCH) and IV (L-CCH). The Type III synthetases (L-Unk) can form labionins. Type IIa D-CCC synthetases have a more highly reactive cyclase, which not only modifies lanthipeptides that chromosomally cluster with the synthetase genes but also modifies lanthipeptides encoded by genes scattered over the genomes of two major ecologically highly significant genera of photosynthetic bacteria, *Chlorococcus* and *Synechococcus* [51,52].

The lanthipeptide synthetase complex comprises domains involved in dehydration and cyclization, sometimes fused in a protein, and sometimes encoded by separate proteins. Similar to other lanthipeptide cluster definition pipelines such as BAGEL [5], we searched for known PFAM [29] domains of cyclase and dehydratases (electronic supplementary material, table S1). In addition, we incorporated searches for an alignment (electronic supplementary material, figure S6) of the dehydratase domain found in Type III/IV synthetases [48].

Of the nine possible combinations of cyclase triads and dehydratases, termed ‘synthetases’ (table 1), five have been described in the literature [53]. While these combinations make up most of the gene clusters in our survey, the predicted clusters also included the four other remaining possible combinations. Two of the four new synthetase combinations have no other known synthetase present in their genome, so these new synthetases are the only obvious route for predicted lanthipeptide production (clustering with peptides T169 and T874 from *Cyanobacteria leptolyngbya* and *Oscillatoria acuminata*; and with T956 and T957 from *Vibrio nigripulchritudo*; electronic supplementary material, dataset 2).

### 3.3. Separation of cyclase clades strongly linked with the three different dehydration mechanisms

To infer an evolutionary tree that explains events at its deep roots, a reliable alignment is required, and the tree should focus on residues that are informative for phylogenetic inference. We selected sequences for alignment and alignment columns for inclusion in the tree, using software visualizations developed to aid in this task (see §2). This selected 389 cyclase sequences for further analysis. This tends to reduce alignment error compared to using the entire dataset, but it is unlikely to eliminate it. Accordingly, bootstrap values for internal edges in the tree (which assume a correct alignment) need to be interpreted with some caution, especially for the deepest branches of the tree. Initial trees along with bacterial proteins indicated that readily alignable eukaryotic cyclase domains from the 2095 eukaryotic species with the LanC-like domain grouped separately from all the bacterial sequences, so only representative eukaryotic sequences are shown here. The tree in figure 1 is arbitrarily rooted with a eukaryotic outgroup. The same tree is illustrated in a rectangular layout with branch lengths drawn proportional to the inferred amount of amino acid change, in electronic supplementary material, figure S1.

The three dehydratases are largely monophyletic (figure 1), with some interesting exceptions. Corroborating the existing literature, where trees were drawn for known cyclases separately, rather than as a single unified tree [23], there is a strong association of the ‘Unk’ cyclases with the lyase dehydration mechanism, and with the labionin motif SxxSx(2–5)C (indicating serine, two non-cysteine residues, serine, two–five non-cysteine residues, cysteine).

The strong association between LanC subtypes and dehydration domains indicates that, in general, once a gene cluster has evolved a dehydration mechanism, it remains with that cluster. There are a few exceptions seen on the tree, where lyase may have replaced LanD or LanB (T107 and T605), or where LanB may have replaced LanD (T334). In some clusters, more than one dehydratase is seen. The fact that two diverse dehydratases exist within a single cluster suggests that they may have differing substrate specificities in lanthipeptide production.

### 3.4. Origins and rates of lanthipeptide evolution

Since all the eukaryotic sequences group distinctly from the bacterial sequences, it may well be that there was an ancient origin of their common ancestor with the bacterial sequences, with the major eukaryotic radiation separating plants and animals occurring over 1000 million years ago [54], but it is not possible to infer at what time point the ability to perform lanthipeptide modification evolved. If the eukaryotic sequences are in fact derived from a bacterial lineage, the bacterial ancestor may be even more ancient. Very low sequence identities observed in a structure-guided alignment [55] of Type I (PDB (Protein Data Bank) [56] code:2g02), Type II (6st5 and 5dzt) and eukaryotic (3t33) cyclases with a farnesyltransferase outgroup (2h6f) suggests that these three cyclase groups diverged in the distant past. The tree derived from this alignment grouped Type I with eukaryotes rather than with Type II, but this did not have significant statistical support from bootstrap analysis. A second insight into the time of origin of the lanthipeptides is suggested by the taxonomic distribution within bacterial phyla.

We grouped the peptides into seven subfamilies that showed greater similarity than expected by chance, even after allowing for their compositional biases in the leader and core peptide regions (justifying a stringent BLAST E-value of 10^−15^, see §2). This identified seven subfamilies (table 2).

**Table 2.** Seven distinct lanthipeptide subfamilies detected within the larger dataset of discovered peptides.

sub family	synthetase^b^		known lantibiotics/lanthipeptides	cysteines	no.	evolutionary rate^a^	distribution	motifs
A	III	L-Unk	erythreapeptin, avermipeptin, stackepeptin, griseopeptin, FlaA, catenulipeptin, SapB	2–3	17	slow	*Actinobacteria*	Labionin, Leader LQ
B	III	L-Unk	—	2	15	slow	*Firmicutes*	Labionin, Leader LQ
C	II/III	D-CCH/ L-Unk	—	1	6	slow	*Actinobacteria (Streptomyces*)	‘reverse- labionin’-like C.S..S
D	I	B-CCH	streptin, nisin U & Z, subtilin, geobacillin I	3–7	33	moderate	*Firmicutes, Actinobacteria*	leader F.L
E	II	D-CCH	—	3–5	5	moderate	*Actinobacteria, Proteobacteria*	
F	IIa	D-CCC	prochlorosin-like	2–3	6	rapid	*Cyanobacteria*	cleavage GG
G	II	D-CCH	macedocin, amylolysin, mersacidin, pneumococcin, haloduracin, lichenicidin	3–5	55	rapid	*Firmicutes*, *Actinobacteria*, *Cyanobacteria*, *Gemmatimonadetes*, *Proteobacteria*	

^a^ See Supplementary figure 2, reflecting the rate of lanthipeptide change relative to change in clustered cyclase.

^b^ Alternative synthetase names correspond to those in table 1.

**Figure 2.**
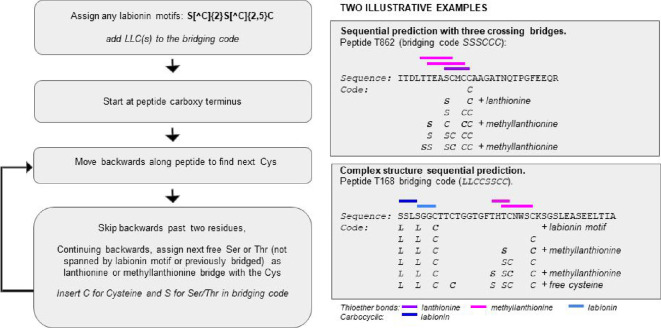
Coding scheme designating predicted lanthipeptide bridging structure. ‘S’ can denote either serine or threonine.

Some lanthipeptide subfamilies showed a high degree of diversification of the core peptide, with similarity depending mainly on the leader sequence. In subfamily G, three core peptides of the thermophilic hydrogen-oxidizing *Kyrpidia tusciae*, and four peptides of the phototrophic cyanobacterium *Stanieria cyanosphaera* (electronic supplementary material, figure S2*g*) showed no similarity to any known core lanthipeptides, which could reflect adaptations to new ecological niches. Similarly, subfamily F has conserved leader peptides [51] but striking core sequence diversity, with most lacking alignment at all 2–3 cysteines (electronic supplementary material, figure S2*f*). This family includes the prochlorosins, whose high promiscuity synthetase substrates include peptides lacking cysteines [57]. Investigated prochlorosins do not appear to have any detectable antimicrobial activity [51], and so may not be a good source of novel lantibiotics.

Peptide subfamily D exhibits diversification of sequence and bridging structure (electronic supplementary material, figure S2*d*), with between three and seven cysteines. While subfamily D includes the known *Firmicutes* (aka *Bacillota*) lantibiotics streptin, nisin, subtilin and geobacillin I, many with a well-conserved CTPxC motif, it also includes a more distant *Actinobacteria* (aka *Actinomycetota*) clade unrelated to known lanthipeptides, with a conserved CxxxCxxxC motif.

The Type III-associated subfamilies A, B and C showed very limited evolutionary diversification of core sequence relative to the rate of change in the cyclase tree (electronic supplementary material, figure S4*a*–*c*). Subfamily C lacks labionin motifs, but instead has the CxSxxS conserved motif; for each of these peptides, there is an additional peptide with no cysteines in the cluster (electronic supplementary material, figure S3), and the cluster includes both Type III (L-lyase) and Type II (D) cyclases, perhaps suggestive of a possible two-peptide complex, with multiple processing steps.

Reconstructing the ancient evolutionary history of lanthipeptide synthesis clusters is complicated by the horizontal transfer of clusters among taxa. Many of the gene clusters are located on plasmids, which facilitate lateral gene transfer of antimicrobial peptides [58–60]. One feature that can be an indicator of long-range horizontal transfer is a difference in G+C contents of the gene of interest and the neighbouring genes of the genome [61]. In general, the G+C content of cyclase genes appeared representative of those found in the rest of the genomes in which they are found, providing no evidence for extensive mobility among distantly related organisms (electronic supplementary material, figure S4). A visualization of 16S gene versus cyclase protein distances highlighted a likely long-range transfer between the phyla actinobacteria and proteobacteria in subfamily E, since the cyclase distances were much lower than expected given the 16S rRNA distances (electronic supplementary material, figure S5).

In contrast with this mobility seen in subfamily E, for some families, pairwise cyclase protein distances among cluster pairs correlated more clearly with the 16S rRNA, in particular for subfamily D. Comparison of cyclase and 16S rRNA trees for subfamily D is consistent with a model where there is no movement of cyclases among phyla, but more frequent movement within phyla (electronic supplementary material S6). The lanthipeptides themselves largely match the cyclase phylogenies, with one or two exceptions (electronic supplementary material, figure S6), but the alignment is not long enough or accurate enough to draw firm conclusions on the phylogenetic grouping. The restriction of cyclase movement among phyla is compatible with two different evolutionary models. In the first scenario, subfamily D originated prior to the divergence of *Firmicutes* and *Actinobacteria*, possibly at least 2 billion years ago [62], and barriers were then erected in both lineages to prevent subsequent transfer (such as dependencies on, or potential for deleterious effects on elements of the core genome). In the second scenario, the cluster evolved in one phylum but was incompatible with most other phyla, for example owing to dependence of synthesis on a host factor. A transfer to the other phylum somehow overcame this barrier, but that adaptation then prevented subsequent back-transfer to the other phylum.

The tree (figure 1) shows three independent bacterial CCC subclades with three zinc binding triad cysteines, each clustering with different dehydratase clades. This extends previous observations which showed that there are two clades of this kind [48]. One of these clades includes the genus *Prochlorococcus*, where the CCC is associated with a more active enzyme and multiple non-clustered peptide substrates [51]. We noted a CCC triad in the eukaryote *Emiliania huxleyii*, contrasting with all other eukaryotic cyclases, which have the CCH triad. While the cyclase of this photosynthetic plankton was difficult to align reliably, it produces large amounts of a simple thioether (diethyl sulphide [63]); its CCC triad could possibly relate to a role in the production of non-peptidic sulphur compounds, or to detoxification of damaged peptides.

### 3.5. Lanthipeptide shared motifs

A SLiMFinder [44] analysis of all predicted peptides revealed an enriched leader LQ motif (VxxLQ) shared across 23 non-redundant groupings of 91 peptides (corrected *p*‐value = 0.006). This motif grouped very strongly with the Type III synthetase, likely enabling synthetase–leader interactions, similar to the known FxLx motif that promotes Type I lantibiotic leader interaction with cyclase [64].

The Type III synthetase clade is clearly associated with predicted labionin motifs (SxxSx{2,5}C, denoted by ‘L’ in figure 1), as expected [11,65]. Four *Streptomyces* peptides (T57, T82, T477, T517) in subfamily C had a conserved motif CxSxxS (see electronic supplementary material, figure S2*c*), suggesting a possible alternative ‘reverse labionin’ substrate. Predicted peptide T358 had a TxxSxxC variant, suggesting an ability of this cyclase to possibly bridge to threonine [66,67]. We noted 46 single cysteine peptides associated with Type III synthetases that lack labionin or reverse-labionin-like motifs. A SLiMFinder search among them for motifs containing only cysteine/serine/threonine revealed five peptides with a labionin-like motif (TxxSx{1,2}C), but the substrate specificity in the remaining 41 peptides may well be less defined. While in general type III synthetases are associated with lower complexity topologies and with fewer cysteines, a few peptides buck this trend, with four Type III associated peptides that lack the labionin motif having four cysteines. These included (electronic supplementary material, dataset 2) one with a repeated 15mer sequence (peptide T77), one with a more complex sequence (T839), one with three repeated ASC motifs (T850) and another that also had an instance of an ASC motif (T929). Additionally, two SC motifs are seen in the two-cysteine peptide T694. Thus, the Type III synthetase may well have specific substrates other than the labionin motif.

Using SLiMFinder [44], we searched across 197 predicted peptides lacking a dehydratase in their cluster, to detect any cysteine-containing motifs for which there is an over-representation of peptides carrying them. A CxxCG motif was significantly more abundant than expected (seen in 18 peptides, corrected *p*‐value (Sig) = 0.03 for enrichment). A total of 55 of the 197 peptides had the simpler CG motif (compared with an expectation of 28 [68]).

### 3.6. Biological limits to the number of bridges and topological complexity

We devised a computational bridging prediction, represented by a summary structure code for each peptide. It is often difficult to precisely order the *in vivo* chemical events that determine peptide modification. Experimental evidence suggests that cyclizations can proceed from a C to N terminal direction, and alternatively in an N to C direction [69]. We investigated whether a simple computational rule could account for most of the known cyclization patterns. The great majority of Lan/MeLan bridges in known lanthipeptides are formed between a cysteine and the preceding unbonded Ser/Thr that is three or more residues away. Following this pattern, we devised a simple prediction algorithm, shown in figure 2.

All cysteines are designated C. Commencing at the carboxy terminus, if the first Cys is part of a labionin motif, indicated by the regular expression S[^C]{2}S[^C]{2,5}C (where ^C denotes any amino acid except cysteine) we denote each of the two serine residues as L for labionin (in practice, the LLC code modification may be either labionin or alternatively modified as a single lanthionine). Otherwise, assign a bridge between cysteine, with the preceding threonine or serine more than two residues away, which we denote as ‘S’. Thus, a peptide with a Lan and a MeLan bridge is coded SCSC if tandemly repeated with uncrossed bridges, or SSCC if they overlap. For the more complex structure shown, the predicted free second cysteine and predicted mixture of labionin and methyllanthionine motifs suggest that the topology is less likely to be an accurate prediction.

**Figure 3.**
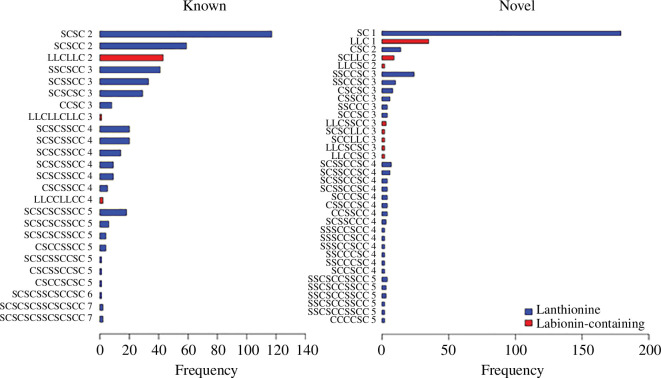
Frequency among non-identical lanthipeptides of predicted bridging topologies. ‘SC’ represents Ser or Thr bridged with Cys, ‘LLC’ represents a labionin motif. See text for detailed description explaining the code. Topologies seen in only one peptide were excluded as they may have a higher frequency of false positives.

This method will not predict nested bridges (seen in sactipeptides, but not in lanthipeptides [70]). Examples of the application of the prediction method to both known and novel peptides are shown in electronic supplementary material, figure S7. For one peptide prediction shown, there are 15 serine or threonine residues and eight cysteines. Without applying any positional preference rules, this yields a total of 15!/(15-8)! theoretically possible bridging combinations, which is in excess of 250 million. The one predicted topology shown is far more likely, as it is consistent with bridging patterns seen in known lanthipeptides. This scheme correctly identified 92 bridging patterns (excluding disulphide bonds) within a dataset of 100 experimentally defined lanthipeptides, so it is not perfect, but the predictions still have some value in making sense of a survey of the diversity of sequences. In our predicted peptide dataset, it indicated 36 suggestively new bridging patterns (observed in two or more lanthipeptides, to reduce false positives that are more likely for suggested bridging topologies). These are additional to the 25 patterns present in known lanthipeptides (figure 3). While known lanthipeptides can have up to seven bridges (in geobacillin I [71] and elgicin [72]), we predicted a *Tannerella forsythia* lanthipeptide with eight predicted bridges (code SCSSCCSCSCSSCCSC, electronic supplementary material, dataset 2). Given the existence of modular tandem duplications in some peptides like this one , the upper limit seen here may be determined by functional utility rather than by synthetic feasibility.

One relatively complex experimentally characterized peptide, geobacillin I, has three bridging overlaps [71]. Our rules (figure 2) represent this as SCSCSCSSCSCSCC; with the rules correctly assuming the bridging of S (Thr or Ser) to Cys according to the following subscripts: S7C7S6C6S5C5S4S3C4S2C3S1C2C1. Myxococin has recently been experimentally characterized as having five overlapping bridges [73]. While our approach predicts five overlaps for this peptide, it assigns them incorrectly. It may be that the predictive power of the algorithm is challenged when presented with more complex structures, which are comparatively rare. The predictions should be viewed as a method to provide indicative suggestions of potential ancient relationships among lanthipeptides lacking sequence similarity, and also a way of summarizing the likely complexity of predicted lanthipeptide libraries.

None of the peptides with predicted crossed bridges involved labionin motifs, suggesting that the Type III cyclase is not capable of generating these. There were numerous predicted crossed bridges for the (methyl)lanthionine topologies. The most topologically complex predicted lanthipeptide in our dataset has five overlaps (SSCCSCSSCSCCC), and a number have four bridging overlaps. Given the theoretical feasibility of a greater number of overlaps, there may be synthetic constraints restricting cyclases from introducing more complex topologies with high efficiency.

A weakness of the predicted novel bridging topologies is that none of the predictions have been validated experimentally. However, we believe this classification is useful in surveying the degree of likely differences in topology within the predicted peptide library. In particular, any peptide that has a very similar cysteine/threonine/serine distribution to a known lanthipeptide may be characterized as lacking apparent topological novelty, even if there is little sequence homology outside of these residues.

### 3.7. Leader sequence properties of experimentally identified lanthipeptides are seen in single-cysteine lanthipeptide predictions

A total of 179 lanthipeptides have a single cysteine, a pattern not previously observed among any of the known lanthipeptides. Labionin-motif containing single-cysteine peptides were enriched in clusters involving Type III synthetases (figure 1), suggesting that they were mainly true-positive predictions. The Me/Lan-containing peptides were seen across both known and novel synthetase types. Among the 179 single-cysteine peptides, 47% had similarity to other single-cysteine peptides in the dataset, compared to only 13% with similarity to precursor peptides with two or more cysteine. Thus, most single-cysteine peptides form a distinctive class, rather than representing degenerate versions of peptides with two or more cysteines. To date, single cysteine lanthipeptides are strongly under-represented among experimentally defined lanthipeptides, with only the further modified labionin lipolanthines known to date [74], so we set out to further investigate whether the 179 predicted single-cysteine peptides share leader peptide properties with experimentally identified lanthipeptides.

Among 94 experimentally defined lanthipeptides, the leaders are hydrophilic and negatively charged, in contrast to their more hydrophobic and positively charged core peptides (electronic supplementary material, figure S8). Eighty-one of the predicted peptides clustered near a C39 peptidase and contained the G[GA] motif, which it cleaves, allowing us to identify likely leader peptides. Overall, there was a strongly correlated amino acid composition of predicted lanthipeptide regions with known lanthipeptide regions (*r* = 0.91, *p* = 10^−8^ for the leader region; *r* = 0.93, *p* = 10^–9^ for the core region), and this pattern was also seen among the subset of single cysteine predicted lanthipeptides (electronic supplementary material, figure S8).

However, for many peptides, the cleavage site is not reliably identifiable. We compared the pattern of amino acid preferences along predicted and known lanthipeptides, calculating the correlation of amino acid presence with residue position, in a 50% redundancy-reduced dataset of 489 peptides, and compared this with a control set of ORFs defined by the same sequence length and compositional rules, but lying outside of lanthipeptide gene clusters (electronic supplementary material, figure S9). Leucine and glutamate were most enriched towards the start of precursors with more than one cysteine, as well as for those with a single cysteine. This indicates that the set of predicted single cysteine lanthipeptides is significantly enriched for the same leader preferences seen in the overall dataset.

Core single-cysteine peptides showed a marked preference for histidine (H, see electronic supplementary material, figure S9). Three Nocardia brasiliensis single-cysteine peptides (T741–T743 electronic supplementary material, dataset 2) have two conserved histidines within the labionin motif (SxxSxHHC), despite being otherwise quite divergent in sequence. There is a sevenfold enrichment of HH dihistidine motifs occurring within the second half of the precursor peptide of single-cysteine peptides, with 11 observed, compared to five in other predicted peptides. This enrichment suggests some potential functionally distinct property of single cysteine peptides, or alternatively some potential impact of the histidines on post-translational modification.

From these investigations of amino acid compositions of leader and core regions of predicted single cysteine lanthipeptides, we conclude that single-cysteine peptides represent an experimentally underinvestigated group of lanthionine cyclase-associated peptides whose functional roles largely remain to be elucidated, but that may not involve antibiotic activity. It is of interest to note that there are a number of predicted single cysteine peptides which are the only candidate peptides in their predicted gene cluster (denoted by filled yellow box, figure 1) and that these are found associated with all three known dehydratases (in figure 2 and zero with lyase, three with B dehydratase, five with D dehydratase, three with both D dehydratase and lyase, 12 with no identified dehydratase). Since there are no single-cysteine lanthipeptides experimentally defined to date, it is clearer to consider the predictions as ‘lanthionine cyclase domain-associated single cysteine predicted peptides’, until some of these predictions are demonstrated experimentally to be modified as lanthipeptides.

## 4. Discussion

In this study, we took a set of complete bacterial genomes and identified a virtual library of predicted lanthipeptide sequences, based on selecting short ORFs with appropriate amino acid composition, that were encoded close to a predicted LanC-like lanthipeptide cyclase protein domain. Associated cyclases and dehydratase domains that were encoded nearby included predicted synthetases involving known and previously unknown combinations of dehydratase and cyclase classes. The set of predicted peptides included some with predicted complex bridging topologies that were apparently novel, as well as an enrichment for single cysteine-predicted peptides. Our approach differs from that of Walker *et al.* [75], who also surveyed incomplete sequence contigs. Their analysis provided strong insights into associated clustered proteins and sequence motifs and an alternative approach to grouping peptides of interest. They did not provide sequence alignments, making it difficult to assess the dynamics of cysteine gain and loss during evolution. They used a machine-learning approach to trim out clusters that did not resemble previously identified lanthipeptides, an approach that would eliminate single cysteine peptides from the dataset, since they are markedly absent from known lanthipeptide training sets. Both approaches have their strengths: while the false-positive rate is reduced in the method by Walker *et al*. [75], the ability to make discoveries beyond what is already known is partially limited. Recognizing the danger of false positives, we carefully inspected both alignments and the statistical properties of the single cysteine peptides, supporting our conclusion that these are an interesting class of predicted peptides worthy of further study.

Our goal in this study was not to compete with the existing BAGEL3 [5] and Walker *et al.*'s [75] approach for the discovery of lanthipeptides with two or more cysteines. Rather, our intention was to survey genomes with a view to deepen our understanding of the overall processes and constraints on lanthipeptide evolution. Those seeking to develop a comprehensive survey of all possible lanthipeptides with two or more cysteines are better directed towards the BAGEL3 [5] and Walker *et al.* [75] approaches which have complementary strengths. Our approach provides some useful guidance for researchers who are focused on a particular biosynthetic cluster, identified through established pipelines, or by other means. Firstly, we recommend that they pay greater attention to considering the functional consequences of any single cysteine peptides encoded in the cluster. Secondly, we suggest a number of approaches to better understand the relationship with lanthipeptides of related function: (i) to employ sequence homology searching versus lanthipeptide libraries but to make sure to apply a stricter similarity threshold than typical, to account for matches determined by the composition rather than the sequence of the precursor peptides, (ii) to identify lanthipeptides with a similar predicted bridging pattern, despite a lack of high sequence similarity, (iii) to identify biosynthetic clusters that are most closely related, by adding their cyclase protein to the curated alignment developed in our study (alignment and tree deposited at https://doi.org/10.5281/zenodo.10779444), to allow inference of a tree topology with statistical support for branchings, and thus gain some insights into the likely evolutionary relationship of the cluster to other clusters.

From their initial evolutionary origin, lanthipeptide synthetases have evolved new modification mechanisms and substrates, resulting in diverse sequences and bridging topologies. Eukaryotic lanCs, which are unassociated with either lanthipeptides or dehydratases, promote thioether bridges of glutathione with dehydrated serine or threonine residues, which can arise through protein damage to phosphorylated sites [76]. From our evolutionary analyses, it was not possible to establish whether the eukaryotic function (damage repair) or the bacterial function (lanthipeptide synthesis) is ancestral.

Type III synthetases showed distinct features in our survey. They do not show evidence of generating crossed bridges in their associated predicted peptides, they are associated with relatively slowly evolving peptide families, and they do not typically undergo lateral gene transfer to distantly related organisms. This combination of features may relate to their function, since Type III peptides typically only possess weak, if any, antibacterial properties, and are known to regulate aerial hyphae formation in *Streptomyces* [21]. The only clade in our survey that has lantibiotic synthetic capacity in all strains investigated is the genus *Streptomyce*s. Across other clades, there is typically a more incomplete or sparse evolutionary distribution of lanthipeptides over the surveyed strains.

The large number and diversity of novel single-cysteine peptides (including both Lan/MeLan, and labionin predictions) is noteworthy, given that lipolanthines are the only experimentally characterized single-cysteine (labionin-derived) lanthipeptide-related lantibiotics [74]. They share similar leader amino acid properties with experimentally identified lanthipeptides (a preference for glutamate and leucine in both sets and a similar charge distribution). While hydrophobicity is often associated with membrane-perturbing antimicrobial activity [77], it is notable that the predicted core single-cysteine peptides are typically less hydrophobic than experimentally characterized lanthipeptides (electronic supplementary material, figure S7). Single-cysteine predicted peptides may play biological roles other than antimicrobial activity, since screening for antimicrobial activity is the dominant mode of experimental discovery of new classes of lanthipeptides [77]. Experimentally characterized lanthipeptides have other functional roles such as signalling, hyphal growth [65], community formation in *Streptococcus* [78] and morphogenetic roles in *Streptomyces* [66,79]. Membrane-disrupting activities of lantibiotics could play non-antimicrobial roles, as seen for *Streptococcus* bacteriocins whose TCS-regulated production increases DNA uptake via competence from surrounding organisms [80,81].

Lantibiotic resistance factors include more generic innate resistance to multiple antibiotics by altering cell wall and membrane [82], as well as more highly specialized resistance factors such as nisinase [83,84], a protease that appears to have evolved a high degree of specificity for lantibiotics. Nisinase-related proteases (MEROPS [85] protease database family S41.UNA) are found in many species and have undergone substantial sequence divergence, but their functions are unknown. Characterization of their functions and phylogenetic distribution may give insights into the evolutionary pressures on lanthipeptides to diversify to evade resistance factors [86].

This study is entirely computational, and while that gives it a wide scope, all the conclusions drawn need experimental validation to support them. We believe that our study represents a useful resource for experimental scientists that will complement existing computational screening tools such as BAGEL3 [5] in providing starting points for thinking about which predicted lanthipeptides are of greatest interest to explore. It would be of great interest to experimentally define the existence and function of some of the single cysteine ORFs and peptides with complex predicted bridging patterns. However, functional assessment of non-lantibiotic lanthipeptides can be challenging: the prochlorosins were identified in 2010, but no function has yet been assigned to them [87]. While culturing strains under appropriate conditions to produce lantibiotics or lanthipeptides may be challenging, heterologous expression systems [88–90] can help overcome these issues. Until the single-cysteine peptides identified in this study have been validated experimentally, we cannot rule out that they may include false positives. Such false positives could arise through not being modified as anticipated, through having additional non-cysteine bridgings introduced via other mechanisms, or possibly even through the generation of interchain bridges to peptides with additional chains.

## Data Availability

Data and code are provided on Zenodo [91] and in the supplementary information [92].
